# Prostate Biopsy in the Case of PIRADS 5—Is Systematic Biopsy Mandatory?

**DOI:** 10.3390/jcm12175612

**Published:** 2023-08-28

**Authors:** Wojciech Malewski, Tomasz Milecki, Stanisław Szempliński, Omar Tayara, Łukasz Kuncman, Piotr Kryst, Łukasz Nyk

**Affiliations:** 1 Second Department of Urology, Centre of Postgraduate Medical Education, 01-809 Warsaw, Poland; wojtek.malewski@gmail.com (W.M.); piotr.kryst@onet.pl (P.K.);; 2 Department of Urology, Poznan University of Medical Sciences, 61-701 Poznan, Poland; 3 Department of Radiotherapy, Medical University of Lodz, 90-419 Lodz, Poland; lukaszkuncman@gmail.com

**Keywords:** fusion biopsy, systematic biopsy, targeted biopsy, prostate cancer, Prostate Imaging Reporting and Data System, biopsy strategy

## Abstract

Combining systematic biopsy (SB) with targeted biopsy (TB) in the case of a positive result from multiparametric magnetic resonance imaging (mpMRI) is a matter of debate. The Prostate Imaging Reporting and Data System (PIRADS) score of 5 indicates the highest probability of clinically significant prostate cancer (csPC) detection in TB. Potentially, omitting SB in the case of PIRADS 5 may have a marginal impact on the csPC detection rate. The aim of this study was to determine whether SB can be avoided in the case of PIRADS 5 and to identify potential factors allowing for performing TB only. This cohort study involved *n* = 225 patients with PIRADS 5 on mpMRI (PIRADS 2.0/2.1) who underwent transperineal or transrectal combined biopsy (CB). CsPC was diagnosed in 51.6% (*n* = 116/225) of cases. TB and SB resulted in the detection of csPC in 48% (*n* = 108/225) and 20.4% (*n* = 46/225) of cases, respectively (TB vs. SB, *p* < 0.001). When the TB was positive, SB detected csPC in *n* = 38 of the cases (38/108 = 35%). SB added to TB significantly improved csPC detection in 6.9% of cases in absolute terms (*n* = 8/116) (TB vs. CB, *p* = 0.008). The multivariate regression model proved that the significant predictors of csPC detection via SB were the densities of the prostate-specific antigen—PSAD > 0.17 ng/mL^2^ (OR = 4.038, 95%CI: 1.568–10.398); primary biopsy setting (OR = 2.818, 95%CI: 1.334–5.952); and abnormal digital rectal examination (DRE) (OR = 2.746, 95%CI: 1.328–5.678). In a primary biopsy setting (*n* = 103), SB detected 10% (*n* = 6/60) of the additional cases of csPC (*p* = 0.031), while in a repeat biopsy setting (*n* = 122), SB detected 3.5% (*n* = 2/56) of the additional cases of csPC (*p* = 0.5). In the case of PSAD > 0.17 ng/mL^2^ (*n* = 151), SB detected 7.4% (*n* = 7/95) of additional cases of csPC (*p* = 0.016), while in the case of PSAD < 0.17 ng/mL^2^ (*n* = 74), SB detected 4.8% (*n* = 1/21) of the additional cases of csPC (*p* = 1.0). The omission of SB had an impact on the csPC diagnosis rate in patients with PIRADS 5 score lesions. Patients who have already undergone prostate biopsy and those with low PSAD are at a lower risk of missing csPC when SB is avoided. However, performing TB only may result in missing other csPC foci located outside the index lesion, which can alter treatment decisions.

## 1. Introduction

Prostate cancer is diagnosed in 1.1 million men annually [1]. Multiparametric magnetic resonance imaging (mpMRI) belongs nowadays to the mainstay diagnostic tools for its management. According to the recommendations of the European Association of Urology (EAU), every biopsy should be preceded by mpMRI [2]. The Prostate Imaging Reporting and Data System version 2 (PIRADS), developed and published in 2015, propelled its use and made its interpretation and reporting uniform [3]. The PIRADS score in current practice is used to stratify the risk before making a definitive decision about biopsy. According to a large Cochrane review, PIRADS scores 4 and 5 are highly suggestive of csPC (clinically significant prostate cancer, defined as International Society of Urological Pathology (ISUP) grade ≥2), with positive predictive values of 48% and 72%, respectively [4]. On the other hand, PIRADS scores 1–2 denote a negative mpMRI result and offer the potential avoidance of prostate biopsy [4].

A prostate biopsy can be performed with a transrectal or transperineal approach. The latest EUA guidelines recommend the transperineal approach over a transrectal approach because of its higher detection rate of csPC and lower infective complications [5]. The fusion of mpMRI and TRUS is the basis of targeted biopsy (TB). MpMRI fusion may be obtained cognitively, with software, or with direct in-bore guidance [6]. TB involves three to five targeted cores obtained from the suspicious area of the prostate gland [7]. The biopsy cores from the rest of the prostate gland are obtained via systematic biopsy (SB). SB involves at least 10 to 12 biopsy cores from both lobes, from apex to base, as far posterior and lateral as possible [8]. The main advantage of TB over SB is the higher detection of csPC and the lower detection of non-significant PC regardless of the biopsy approach (transrectal vs. transperineal) [9]. However, the EAU guidelines recommend a combined biopsy (CB), including TB and SB, in the case of positive mpMRI results [2]. The benefits of additive SB are still not clear. The arguments for CB include better tumor grading and compensation for targeting errors or PC lesions invisible in mpMRI. According to the EAU guidelines, TB may be performed without SB in a repeat biopsy setting [2]. SB alone is still recommended in the case of a negative PIRADS score (1–2) and a high suspicion of prostate cancer, such as when there is a high PSA/PSAD (prostate-specific antigen/density of prostate-specific antigen) or abnormal DRE (digital rectal examination) [4].

There is growing interest in diagnosing csPC with a low number of biopsy cores. Limiting the number of biopsy cores decreases pain, which is cumulative in nature [10]. Moreover, even in the setting of a transperineal biopsy, the number of biopsy cores has proven to influence infective complications [11]. As for the more obsolete transrectal biopsy, urinary tract infection and prostatitis are much more common in 18-core biopsy than in 12-core biopsy [12]. Decreasing the number of biopsy cores may also limit bleeding complications from the procedure, including bladder tamponade [13]. Interestingly, extensive biopsies may impact the course of radical prostatectomy and result in higher blood loss [14]. The omission of SB would significantly reduce the number of biopsy cores. On the other hand, SB may still contribute to the diagnosis of a non-negligible number of csPC cases, and omitting it may lead to misdiagnosis [9].

The aim of this study was to determine whether SB can be avoided in PIRADS 5 and to identify potential factors that would allow performing TB only. The outcomes of this study were the rates of csPC detection in SB, TB, and overall (CB = SB + TB).

## 2. Materials and Methods

### 2.1. Materials and MRI–Ultrasound Fusion Biopsy

This retrospective study involved 225 patients with PIRADS 5 on mpMRI (PIRADS 2.0/2.1, images from external radiological centers) who underwent transperineal or transrectal CB using the Trinity Koelis^®^ navigation system (Koelis, Meylan, France) under local anesthesia. Biopsies involved at least three targeted cores from the mpMRI lesion and ten to twelve systematic cores from non-targeted areas. Procedures were performed under local anesthesia by a single experienced operator. If more than one lesion was present at mpMRI, the index lesion was defined as one having the highest PIRADS assessment category or as the largest lesion in the case of there being more than one within the same category. CsPC was defined as a Gleason score of 3 + 4 or higher (ISUP grade ≥2).

### 2.2. Statistical Analysis

Non-normally distributed continuous variables were reported as medians (Me) with the interquartile range (IQR) and compared using the Mann–Whitney U test. PSAD (density of prostate-specific antigen) was calculated using PSA divided by the MRI-derived prostate volume (ellipsoid method). The receiver operating characteristic (ROC) curves for diagnosis of csPC via PSAD were analyzed. Youde’s index (sensitivity + specificity-1) for identification of the optimum cut-off point for PSAD was used as a predictor of csPC detection. Categorical variables were reported as frequencies and proportions. Differences in rates were tested using the chi-squared test or McNemar test. Univariable and multivariable regression models were performed to evaluate predictors of csPC detection in SB and TB. Odds ratio (OR), 95% confidence interval (95%CI) of the odds ratio, and *p* values were recorded. A *p* value of <0.05 was considered significant. SPSS© software (SPSS statistics 25) was used for statistical analysis.

## 3. Results

We retrospectively analyzed the total number of *n* = 794 patients who underwent MRI ultrasound fusion biopsies of their prostate at the ECZ Hospital Otwock, Poland, between November 2016 and June 2021. Data were collected from medical patient records, which included the following: age, previous medical history, pre-biopsy PSA, PSAD, MRI report, biopsy procedure report, and pathology report.

### 3.1. Characteristics of the Group and Comparison of Clinical Data between the csPC Group and No-csPC Group

**Figure 1:**
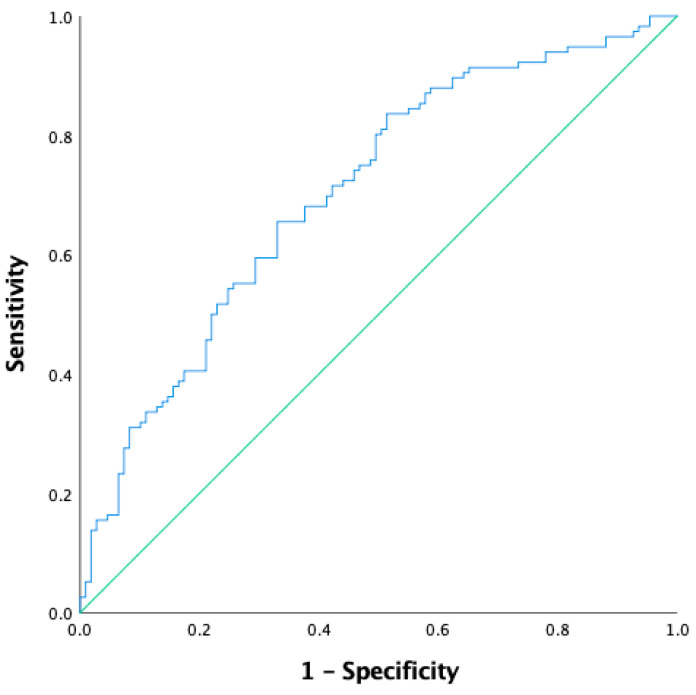
The receiver operating characteristic (ROC) curve for diagnosis of clinically significant prostate cancer (csPC) via PSAD (density of prostate-specific antigen). AUC–0.704 (95%CI: 0.636–0.771), *p* < 0.001.

The total number of *n* = 225 men with a PIRADS score of 5 who underwent CB were included in this study. CB detected *n* = 116/225 (52%) cases of csPC. Patients with csPC significantly differed statistically from those without csPC in the following parameters: PSA level—10.5 ng/mL (6.0–15.0) vs. 7.6 ng/mL (5.0–11.1) (*p* < 0.001); PSAD—0.25 ng/mL^2^ (0.16–0.45) vs. 0.15 ng/mL^2^ (0.10–0.23) (*p* < 0.005); prostate volume—38.8 mL (31.6–49.5) vs. 48.0 mL (37.0–66.0) (*p* = 0.048); and abnormal DRE: *n* = 50/116 (43.1%) vs. normal DRE *n* = 23/109 (21.1%) (*p* < 0.05). There were no differences between the above groups in terms of age (*p* > 0.05); biopsy variant—transperineal vs. transrectal (*p* = 0.5); the zone location of the dominant lesion—peripheral vs. non-peripheral (*p* = 0.05); or biopsy history—primary vs. repeat (*p* = 0.07). The results are presented in Table 1. The AUC for diagnosis of csPC via PSAD was 0.704 (95%CI: 0.636–0.771, *p* < 0.001) (Figure 1). The highest Youden’s index was at a PSAD level of 0.17 ng/mL^2^. At this point, the diagnosis of csPC had 68% sensitivity and 61% specificity.

### 3.2. MRI Targeted Biopsy vs. Combined Biopsy

We analyzed the detection rate of csPC by the type of biopsy performed, i.e., TB, SB, or CB. csPC was detected via TB in 48.0% (*n* = 108/225) of cases, via SB in 20.4% (*n* = 46/225) of cases (SB vs. TB, *p* < 0.001), and via CB in 52.0% (*n* = 116/225) of cases. When TB was positive (*n* = 108), SB detected csPC in 35% of cases (*n* = 38/108). SB added to TB significantly improved csPC detection in the absolute number of 6.9% csPC cases (*n* = 8/116) (TB vs. CB, *p* = 0.008).

### 3.3. Clinical Factors Impacting the Detection of csPC in SB and TB

We analyzed which clinical factors impact the risk of csPC detection separately for SB and TB. For SB, the multivariate regression model proved that the following clinical parameters were significantly increasing the probability of csPC detection: PSAD > 0.17 ng/mL^2^ (OR = 4.038; 95%CI: 1.568–10.398) primary biopsy setting (OR = 2.818; 95%CI: 1.334–5.952), and abnormal DRE (OR = 2.746; 95%CI: 1.328–5.678). For TB, a multivariate regression model provided that the following clinical parameters were increasing the probability of csPC detection: PSAD > 0.17 ng/mL^2^ (OR = 3.759; 95%CI: 2.002–7.060) and abnormal DRE (OR = 2.911; 95%CI: 1.576–5.377). The biopsy-naive status was not a significant predictor of csPC detection in TB (OR = 1.388; 95%CI: 0.820–2.349). The results are presented in Table 2.

### 3.4. Role of Clinical Factors: PSAD and Primary vs. Repeat Biopsy for csPC Detection in SB

Clinical parameters, primary vs. repeat biopsy and PSAD (low vs. high), were analyzed to assess the csPC detection rate in SB, TB, and CB and the risk of missing csPC when SB is omitted. The results are presented in Table 3.

#### 3.4.1. Biopsy Setting—Primary vs. Repeat

In a cohort of biopsy-naive patients *n* = 103, csPC was diagnosed in 58.3% (*n* = 60/103) of the cases via CB. SB detected 30.0% (*n* = 31/103) of csPC cases, and TB detected 52.4% (*n* = 54/103) of csPC. SB added to TB significantly improved csPC detection in the absolute number of 10% (*n* = 6/60) of csPC cases (*p* = 0.031).

In a cohort of *n* = 122 patients with repeat biopsy, csPC was diagnosed in 45.9% (*n* = 56/122) of the cases via CB. SB detected 12.3% (*n* = 15/122) of csPC cases, and TB detected 44.3% (n = 54/122) of the csPC cases. SB added to TB improved csPC detection in the absolute number of 3.6% (*n* = 2/56) of the csPC cases (*p* = 0.5).

The detection of csPC via SB in the biopsy-naive cohort was significantly higher than in the cohort with repeat biopsy (30.0% vs. 12.3%; *p* = 0.001). Omitting SB in the primary biopsy is associated with a higher risk of missing csPC than in the cohort with previous biopsy (10% vs. 3.6%; *p* = 0.09).

#### 3.4.2. Low PSAD (<0.17 ng/mL^2^) vs. High PSAD (>0.17 ng/mL^2^)

In a cohort of *n* = 74 patients with low PSAD (<0.17 ng/mL^2^), csPC was diagnosed in 28.4% (*n* = 21/74) of the cases via CB. SB detected 8.1% (*n* = 6/74) of csPC cases, and TB detected 27.0% (*n* = 20/74) of the csPC cases. SB added to TB improved csPC detection in the absolute number of 4.8% (*n* = 1/21) of csPC cases (*p* = 1.0).

In a cohort of *n* = 151 patients with high PSAD (>0.17 ng/mL^2^), csPC was diagnosed in 62.9% (*n* = 95/151) of the cases via CB. SB detected 26.5% (*n* = 40/151) of csPC cases, and TB detected 58.3% (*n* = 88/151) of the csPC cases. SB added to TB significantly improved csPC detection in the absolute number of 7.4% (*n* = 7/95) of csPC cases (*p* = 0.016).

The detection of csPC via SB in the cohort with high PSAD was significantly higher than in the cohort with low PSAD (26.5% vs. 8.1%; *p* < 0.001). Omitting SB in the cohort with high PSAD values is associated with a higher risk of missing csPC than in the cohort with low PSAD values (7.4% vs. 4.8%; *p* = 0.21).

## 4. Discussion

The main goal of our study was to assess whether omitting SB in PIRADS 5 lesions could impact the csPC detection rate. Our results confirmed that in PIRADS 5, TB has a significantly higher csPC detection rate compared to SB, 48% vs. 20%, respectively. We also confirmed that omitting SB may contribute to a significant reduction in the csPC detection rate by 6.9%. Our results are consistent with data from recent studies. Ahdoot et al. found that TB-only biopsy misses 5.8% of csPC [15]. In a Cochrane meta-analysis, the added value of SB in csPC detection in a biopsy-naive setting was 4.3% [9]. Similarly, in both the 4M trial and MRI-FIRST, the added value of systematic biopsy was estimated at a level of 5% [16,17]. Porpiglia et al. compared the detection rate of csPCa between TB alone and TB combined with SB. This non-inferiority designed study concluded that TB alone was not inferior to the fusion biopsy combined with SB for the detection of csPCa [18]. However, the above results apply to all mpMRI-positive men. The PIRADS 5 score is particularly associated with the highest probability of detecting csPC in TB. Recent studies have confirmed that in PIRADS 5, the risk of missing csPC with the TB-only approach may be even lower than in PIRADS 3 and 4 [19,20,21,22,23,24]. In a study by Nakanishi et al., TB only missed 4.6% of csPC in PIRADS 5 patients vs. 22% for the remaining men with PIRADS 3 and 4 scores [19]. Similarly, in another study by Gomez et al., SB improved csPC detection in PIRADS 3 and 4 patients by 26.3% and 9.5%, respectively, but there was no improvement in PIRADS 5 patients [20]. In the next study, Drobish et al. demonstrated that in PIRADS 5 patients, there would be no improvement in csPC detection when SB was omitted [21]. Additionally, in another study by Tafuri et al., only 4% of csPC was missed when omitting SB in PIRADS 5 patients [22].

The results from our study and those cited above indicate that concomitant SB has a marginal impact on csPC detection in PIRADS 5 patients. However, on the other hand, there are several arguments that advise against SB omission. First of all, we should take into consideration the limited sensitivity of mpMRI in detecting all csPC foci, as approximately 30% of csPC is invisible on mpMRI [25]. Moreover, prostate cancer is characterized by multifocal growth, which concerns about 20% of cases [26]. In the study by Checcucci et al., prostate cancer was detected via concomitant SB contralaterally to index lesions in 36% of csPC cases detected via TB [27]. It has also been proven that a higher PIRADS score increases the likelihood of csPC presence outside the index lesion [28]. In PIRADS 5 patients, csPC presence outside the index lesion can be seen in up to 60% of cases [29]. This relationship was also confirmed in our study, as SB detected csPC outside the index lesion in 35% of cases.

In addition to the PIRADS score, there are also other potential predictors of csPC detection via SB which are under investigation, such as PSA, PSAD, biopsy setting (primary vs. repeat), DRE status, lesion location, and prostate volume. However, any risk-adapted strategy or nomogram has already been proposed to avoid concomitant SB. In our study, we confirmed that the risk of csPC detection in SB is significantly lower in repeat biopsy than in primary biopsy by 12.3% vs. 30.0%, respectively. We found that SB added to TB in the primary biopsy significantly improved csPC detection in contrast to the repeat biopsy. The risk of missing csPC when SB is omitted is lower in repeat biopsy in comparison to primary biopsy by 3.5% vs. 10%, respectively. Our results reflect the data available in other publications. Extercate et al. detected only 1.3% of csPC cases via SB in repeat biopsies [30]. A Cochrane meta-analysis proved that the added value of SB in repeat biopsy is low, with estimates of 2.3% [9]. The above and our results are consistent with EAU recommendations, which state that SB may be omitted in repeat biopsy; however, it should be noted that the amount of evidence for this recommendation is weak [2].

In our work, we also confirmed that PSAD is another significant predictor increasing the detection rate of csPC in PIRADS 5 patients. We confirmed that a PSAD level of 0.17 ng/mL^2^ is optimal for detecting csPC. The results of our analysis are consistent with the available literature, which indicate that PSAD and PIRADS scores are complementary in the detection of csPC. In a clinical scenario, the PSAD value may influence the decision to perform a prostate biopsy [31]. For example, in non-suspicious mpMRI results, it is recommended to perform SB at high PSAD values (>0.15 ng/mL^2^) because the risk of csPC detection is significantly increased [32]. An analogous strategy is also adopted in patients with intermediate lesions, i.e., PIRADS 3, where CB should be performed in high PSAD [32]. Additionally, in PIRADS 4 and 5 patients, the impact of PSAD on the csPC detection rate has been demonstrated [33]. The results of our analysis confirmed that omitting SB at high PSAD values increases the risk of missing csPC from 4.7% to 7.4%. The absolute detection rate of csPC in SB was increased from 8.1% to 26.5% for the PSAD 0.17 ng/mL^2^ threshold. Recent publications based on a comparison of histopathology reports from a biopsy and radical prostatectomy indicate that high PSAD values are also associated with higher tumor volume and a higher probability of underestimating the Gleason score [34,35]. It should, therefore, be concluded that in high PSAD values, SB should not be omitted in PIRADS 5 lesions.

Our results confirmed that clinical data such as PSAD and biopsy settings are significant predictors of csPC detection via SB. However, in our study, we did not analyze the effects of omitting SB and clinical predictors on the underestimation of the Gleason score, and this is also a clinically relevant issue considering the justification for performing SB. Several recent studies indicate that SB may reduce the likelihood of Gleason score underestimation [36,37]. Moreover, taking cores from areas surrounding a suspicious lesion (focal systematic biopsy) can overlap sampling error and may provide a better estimation of the Gleason score [38]. Similarly, several technical issues of TB are also under investigation. For example, the number of targeted cores taken per lesion may impact csPC detection and Gleason score estimation [39]. In particular, obtaining three/four cores (current standard) from high-volume lesions, such as PIRADS 5, may not be sufficient to adequately estimate the Gleason score [40]. Moreover, mpMRI alone has a limited ability to predict local stage and extra-prostatic extension [41,42]. As a result, a TB-only approach may impact clinical decisions, such as qualification for local treatment or planning the extension of surgical intervention. For example, the presence of a PC outside index lesion should preclude focal treatment [43]. The local stage of prostate cancer also provides implications regarding neurovascular bundle sparing during radical prostatectomy [44]. It is also worth noting that the percentage of PC involvement in the systematic cores is an important prognostic factor, indicating the risk of biochemical progression or pelvic lymph node involvement [45,46]. In conclusion, the omission of SB may have a negative impact on adequate PC local staging and prognostic group determination.

### Limitations

Our study was single-center and retrospective. We did not verify the histopathological report from the radical prostatectomy. The study population was heterogeneous, as it included both transperineal and transrectal biopsies. The analysis did not include epidemiological factors such as body mass index (BMI) or comorbidities. Recently, BMI was proven to correlate with the detection of high-grade prostate cancer in biopsies [47]. In this regard, comparing such factors with the potential omission of systematic biopsy would be very interesting. The mpMRI images were obtained from external radiological centers, and they were described by radiologists with different experiences; therefore, there may have been certain discrepancies in the assessment of the PIRADS scores. In our study, we did not analyze the effects of omitting SB on the underestimation of the Gleason score. Comparisons in patients with multiple MRI suspicious lesions were not available in this study. The comparisons of biopsy methods were performed per patient rather than per lesion.

## 5. Conclusions

The results of our study indicate that in PIRADS 5 lesions, the probability of detecting csPC in SB is significantly lower than in TB (20% vs. 48%, respectively). Omitting SB is also associated with the risk of missing csPC in 6.9% of cases. SB, however, contributed to the detection of distinct csPC foci in 35% of patients with csPC diagnosed simultaneously via TB. The omission of SB may impact local staging by failing to detect multifocal csPC and thus may affect therapeutic decisions. We also showed that significant predictive factors increasing the risk of csPC detection outside the index lesion via SB include high PSAD (>0.17 ng/mL^2^) and primary prostate biopsy. Our study suggests that SB might be omitted in patients with PIRADS 5 scores and low PSAD or secondary biopsy settings. However, further prospective studies, especially those correlating biopsy results with whole-mount radical prostatectomy specimens, are needed to set firm recommendations.

## Figures and Tables

**Table 1:** Descriptive characteristics. (csPC—clinically significant prostate cancer; PSA—prostate-specific antigen; PSAD—density of prostate-specific antigen; DRE—digital rectal examination; mpMRI—multiparametric magnetic resonance imaging; Me—median; IQR—interquartile range; *n*—number of cases).

	Total (*n* = 225)	No csPC (*n* = 109)	csPC (*n* = 116)	No csPC vs. csPC *p* Value
Age (years) Me (IQR)	67.0 (62.0–70.0)	67.0 (62.0–70.0)	67.0 (62.0–71.0)	*p* > 0.05
PSA (ng/mL) Me (IQR)	8.6 (5.6–14.0)	7.6 (5.0–11.1)	10.5 (6.0–15.0)	*p* < 0.001
PSAD (ng/mL^2^) Me (IQR)	0.19 (0.13–0.35)	0.15 (0.10–0.23)	0.25 (0.16–0.45)	*p* < 0.005
DRE *n* (%):				*p* < 0.05
Normal	147 (65.3%)	83 (76.1%)	64 (55.2%)	
Abnormal	73 (32.4%)	23 (21.1%)	50 (43.1%)	
No info	5 (2.2%)	4 (3.7%)	2 (1.7%)	
Biopsy *n* (%):				*p* = 0.07
1—primary	103 (45.8%)	43 (39.4%)	60 (51.7%)	
>1—repeat	122 (54.2%)	66 (60.6%)	56 (48.3%)	
Prostate volume (mL) Me (IQR)	42.0 (33.0–60.0)	48.0 (37.0–66.0)	38.8 (31.6–49.5)	*p* = 0.048
Max diameter of IL in mpMRI (mm) Me (IQR)	19.0 (16.0–23.0)	18.0 (16.0–23.0)	19.0 (23.0–24.0)	*p* > 0.05
mpMRI zone location *n* (%):				*p* = 0.05
Peripheral zone	145 (64.4%)	62 (56.9%)	83 (71.6%)	
Non-peripheral zone	51 (22.7%)	30 (27.5%)	21 (18.1%)	
No information	29 (12.9%)	17 (15.6%)	12 (10.3%)	
Biopsy access *n* (%):				*p* = 0.5
Transrectal	53 (23.6%)	26 (23.9%)	27 (23.3%)	
Transperineal	172(76.4%)	83 (76.1%)	89 (76.7%)	

**Table 2:** Univariate and multivariable analysis of the clinical factors predictive for clinically significant prostate cancer detection (csPC) using systematic biopsy (SB) and targeted biopsy (TB). PSA—prostate-specific antigen; PSAD—density of prostate-specific antigen; DRE—digital rectal examination; mpMRI—multiparametric magnetic resonance imaging; OR—odds ratio; CI—confidence interval; ref—reference.

	Univariate Analysis		Multivariate Analysis	
Systematic Biopsy				
	**OR (95%CI)**	***p*** **Value**	**OR (95%CI)**	***p*** **Value**
Age (years) (lineal)	0.984 (0.943*–*1.025)	0.436		
PSA (ng/mL) (lineal)	1.013 (1.000*–*1.027)	0.058		
DRE: normal (ref) abnormal (1)	3.509 (1.773*–*6.944)	0.001	2.746 (1.328*–*5.678)	0.008
PSAD: <0.17ng/mL^2^ (ref) >0.17ng/mL^2^ (1)	4.084 (1.645*–*10.14)	0.002	4.038 (1.568*–*10.398)	0.004
Prostate volume (mL): 0*–*30 mL (ref) 30*–*60 mL (1) >60 mL (2)	0.932 (0.397*–*2.186) 0.306 (0.093*–*1.006)	0.871 0.051		
Index lesion diameter (mm) (lineal)	1.043 (0.995*–*1.093)	0.082		
Biopsy: Repeat (ref) Primary (1)	3.071 (1.548*–*6.093)	0.001	2.818 (1.334*–*5.952)	0.006
**Targeted Biopsy**				
Age (years) (lineal)	1.022 (0.987*–*1.058)	0.216		
PSA (ng/mL) (lineal)	1.035 (1.008–1.063)	0.11		
DRE: normal (ref) abnormal (1)	2.864 (1.595*–*5.141)	0.001	2.911 (1.576*–*5.377)	0.001
PSAD: <0.17ng/mL^2^ (ref) >0.17ng/mL^2^ (1)	3.771 (2.056*–*6.917)	0.001	3.759 (2.002*–*7.060)	0.001
Prostate volume (mL): 0*–*30 mL (ref) 30*–*60 mL (1) >60 mL (2)	0.747 (0.355*–*1.571) 0.357 (0.149*–*0.854)	0.441 0.21		
Index lesion diameter (mm) (lineal)	1.023 (0.982*–*1.066)	0.272		
Biopsy: Repeat (ref) Primary (1)	1.388 (0.820*–*2.349)	0.222		

**Table 3:** Comparison of clinically significant prostate cancer (csPC) detection and missed rate between targeted biopsy (TB) alone, systematic biopsy (SB) alone, and combined biopsy (CB) in the whole cohort; in primary vs. repeat biopsy setting; and in low PSAD vs. high PSAD level. csPC—clinically significant prostate cancer; TB—targeted biopsy; SB—systematic biopsy; CB—combined biopsy; PSAD—density of prostate-specific antigen; *n*—number of cases.

	SB *n* (%)	TB *n* (%)	CB *n* (%)	TB vs. CB *p* Value
	All biopsies *n* = 225			
csPC detection	46/225 (20.4%)	108/225 (48%)	116/225 (51.5%)	0.008
csPC missed	70/116 (60.3%)	8/116 (6.9%)	-	
No csPC	-		109/225 (48.4%)	
	**Initial biopsy *n* = 103**			
csPC detection	31/103 (30.0%)	54/103 (52.4%)	60/103 (58.3%)	0.031
csPC missed	29/60 (48.3%)	6/60 (10.0%)	-	
No csPC	-		43/103 (41.7%)	
	**Repeat biopsy *n* = 122**			
csPC detection	15/122 (12.3%)	54/122 (44.3%)	56/122 (45.9%)	0.5
csPC missed	41/56 (73.2%)	2/56 (3.6%)	-	
No csPC	-		66/122 (54.1%)	
	**PSAD < 0.17 ng/mL^2^ *n* = 74**			
csPC detection	6/74 (8.1%)	20/74 (27.0%)	21/74 (28.4%)	1.0
csPC missed	15/21 (71.4%)	1/21 (4.8%)	-	
No csPC	-		53/74 (71.6%)	
	**PSAD > 0.17 ng/mL^2^ *n* = 151**			
csPC detection	40/151 (26.5%)	88/151 (58.3%)	95/151 (62.9%)	0.016
csPC missed	55/95 (57.9%)	7/95 (7.4%)	-	
No csPC	-		56/151 (37.1%)	

## Data Availability

The data analyzed in this study are available upon request from the corresponding author.
